# Perspectives of Patients With Relapsed and Refractory Acute Myeloid Leukemia and High-Risk Myelodysplastic Syndrome on Patient–Clinician Communication About Prognosis and the Future

**DOI:** 10.1089/pmr.2023.0064

**Published:** 2024-01-19

**Authors:** Vinay Rao, Sarah Linsky, M. Tish Knobf

**Affiliations:** ^1^ Division of Palliative Medicine, Department of Medicine, Yale University School of Medicine, New Haven, Connecticut, USA.; ^2^ School of Nursing, Yale University, Orange, Connecticut, USA.

**Keywords:** communication, future, leukemia, prognosis, uncertainty

## Abstract

**Background:**

Patients with acute myeloid leukemia (AML) and high-risk myelodysplastic syndrome (MDS) experience unpredictable disease trajectories and high prognostic uncertainty, which serve as barriers to patient–clinician communication about prognosis and their values and preferences for the future in the event of worsening health. Little is known about patients' day-to-day lived experiences and how this shapes their willingness to engage in such conversations.

**Objectives:**

To explore participant perspectives on living with their illness and patient–clinician communication about prognosis and the future.

**Design:**

This is a qualitative study using semi-structured interviews.

**Setting/Subjects:**

Patients with relapsed and refractory (R/R) AML and high-risk MDS from a northeastern U.S. cancer center.

**Data Collection:**

Interviews were transcribed verbatim and thematic analysis was used to generate findings.

**Results:**

Of the 14 participants, the mean age was 66 years, 79% were men, 93% were White, married, and had AML. The overarching theme that describes the experience was *“Taking One Day at a Time” in a Fog of Uncertainty.* Uncertainty was a universal perception related to the challenges for clinicians to predict prognosis. To cope with uncertainty, most participants tried to focus on the present and maintain normality in everyday life. Participants valued encouragement and positivity in patient–clinician communication, however, the majority were not ready to discuss prognosis and the future in the event of worsening health. Of note, 7 of 14 participants died within three months after the interview.

**Conclusions:**

These data describe a unique perspective of patients with R/R AML and high-risk MDS that clinicians could use to enhance communication strategies.

## Introduction

Patients diagnosed with acute myeloid leukemia (AML) and high-risk myelodysplastic syndrome (MDS) experience prognostic uncertainty and low survival rates.^1^ At the end of life, these patients have high rates of health care utilization and low rates of hospice enrollment, in part related to late initiation or lack of goals-of-care discussions.^1–4^ Some clinicians are hesitant to talk about death and dying because they fear it will diminish patient trust, take away hope, and cause unnecessary psychological harm.^5^ Yet, patient–clinician communication about goals of care can improve the quality of end-of-life care for patients.^6^

Palliative care is a medical specialty that helps patients with serious illness live well despite their disease. An important component of palliative care is serious illness communication, which describes patient–clinician conversations designed to assess patient illness understanding, discuss prognosis, and explore values and preferences for future care in the event of worsening health.^6–8^ This approach to communication can help patients with life-limiting illnesses feel like their voices are being heard, assist with medical decision making, help patients process their emotions, and reduce psychological distress.^6,7^

Prior studies using a structured tool to facilitate serious illness communication in clinical practice showed improved outcomes for patients with predominantly incurable solid tumors.^7^ However, patients with AML and high-risk MDS are a unique population that experience unpredictable illness trajectories, high prognostic uncertainty, and low prognostic awareness, which may shape their willingness to discuss certain topics with their clinicians.^1,7,9^

In a study evaluating older patients with AML and MDS, although most felt that a structured serious illness conversation tool could improve their illness and prognostic understanding, they also wanted their clinicians to use hope and positivity even when prognosis was poor.^10^ This highlights the inherent challenges to engaging patients with AML and MDS in discussing prognosis and their values and preferences for future and end-of-life care.

There is a gap in the literature in understanding the day-to-day experience of patients with AML and high-risk MDS and the way this shapes their willingness to discuss prognosis and the future with their clinicians. By learning about the patient experience in greater depth, these perspectives can help us tailor and adapt patient–clinician communication approaches.

## Materials and Methods

The purpose of this qualitative study was to describe the perspectives of patients with relapsed and refractory (R/R) AML and high-risk MDS on patient–clinician communication, specifically discussions about treatment options and prognosis, how they coped with the uncertainty of their prognosis, and their feelings about discussing the future in the event of worsening health.

This was an exploratory descriptive study using qualitative inquiry to understand patient–clinician communication from the perspective of the participants that is grounded in the reality of their daily lives.^11^ The study was guided by interpretive description, which is a pragmatic qualitative approach in health research to address complex clinical phenomena.^12^ After IRB approval, from July 2022 to January 2023, four oncologists within a northeastern U.S. cancer center referred participants who met the inclusion criteria (age ≥18 years, diagnosed with R/R AML or high-risk MDS, receiving cancer-directed treatment, English speaking, and have medical decision-making capacity) to the study.

**FIG. 1.**
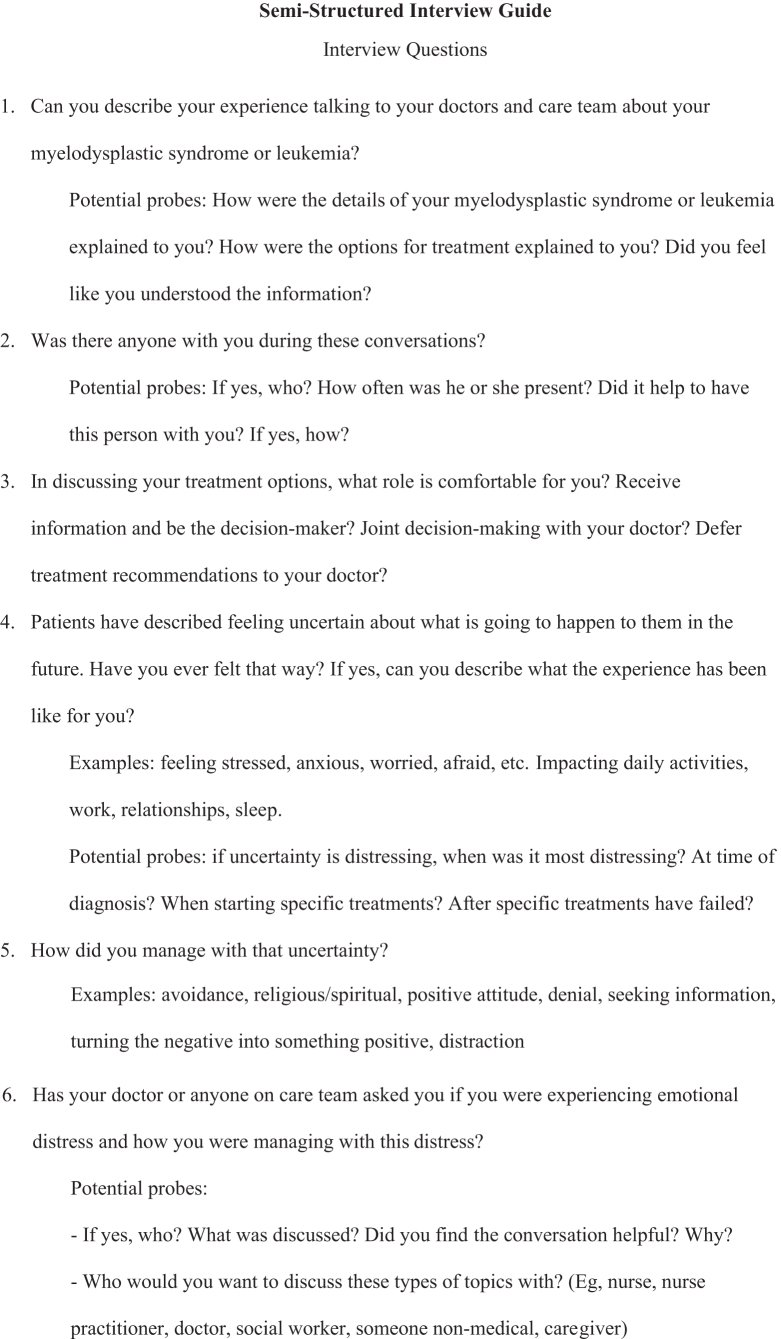

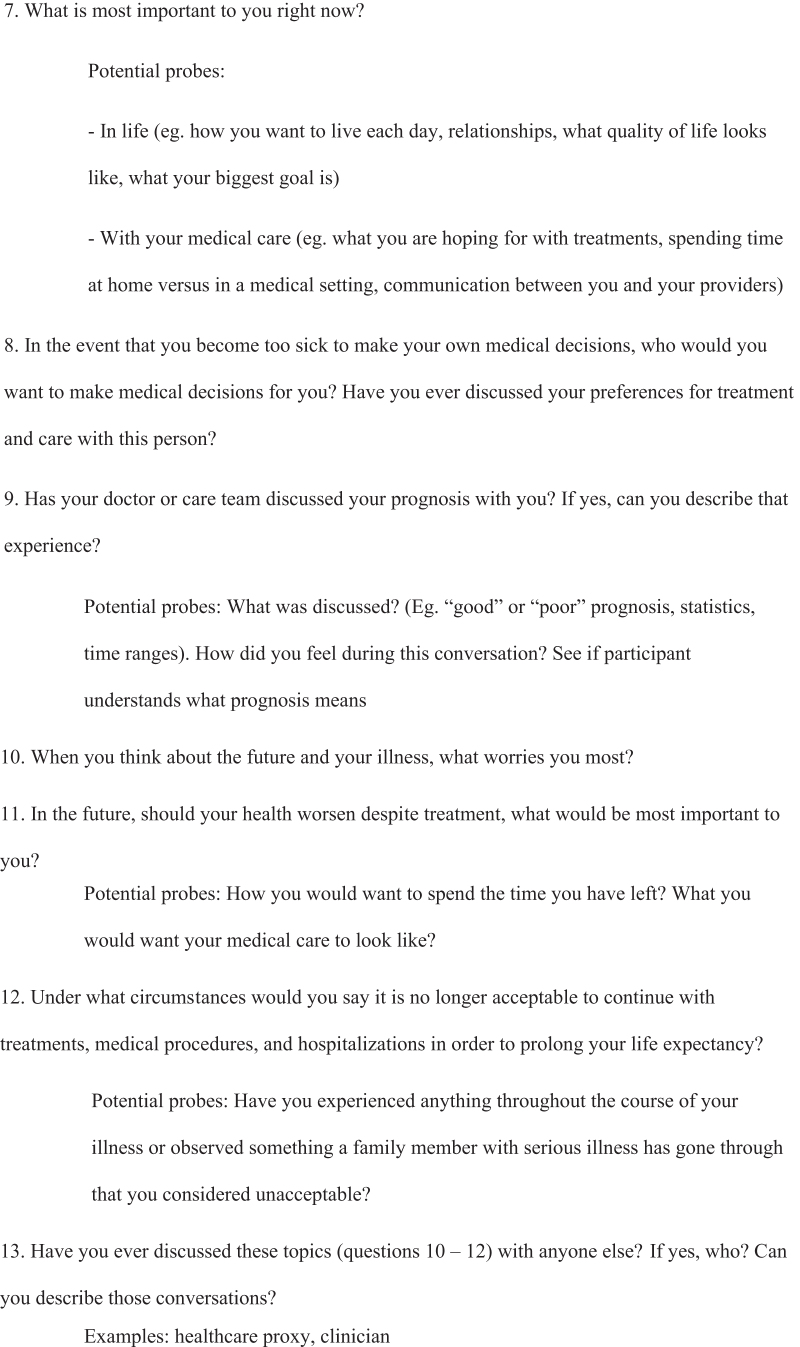

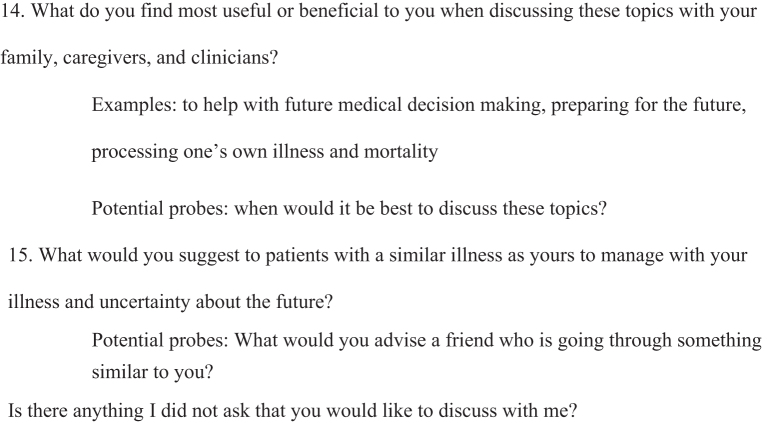
Semistructured interview guide questions.

We excluded patients with higher chance of cure (absence of R/R disease) based on the preferences of our referring oncologists, since some of our interview questions discussed the possibility of dying. We also excluded patients receiving best supportive care and/or hospice. All interviews were conducted either in person or over videoconferencing by one team member with expertise in qualitative research (S.L.). Verbal informed consent was obtained to participate in a recorded interview. A semi-structured interview guide (Fig. 1) was designed to capture participant perspectives. Interviews were audiorecorded and sent to a HIPAA-compliant transcription service.

Transcripts were read in entirety by all three authors.^11,13^ One author (M.T.K.) was responsible for the initial inductive analysis beginning with open and *in vivo* coding.^13^ Provisional codes were examined for redundancy and overlap and were then clustered and collapsed into themes.^12,14^ Analytic memos were recorded throughout and integrated into analysis. Using a team-based approach to analysis, data were formatted into tables for review of the meaning of codes, emerging themes, and representative quotes.^15^ During the synthesis phase of analysis, visual displays were created to support interpretation of the data.^11^ The iterative process of synthesis, abstraction, and interpretation provided the confidence that rich meaningful conclusions were reached that were consistent with the purpose of the study.^16,17^

## Results

The sample consisted of 14 participants. A relatively narrow study aim, homogeneous population, and seasoned researcher in qualitative analysis reflect a sample size sufficient to achieve information power and meaning saturation of the data.^17–19^

Of the 14 participants, the mean age was 66 years old, 79% were men, 93% were White and married, and 72% had college education or higher (Table 1). Diagnoses included 10 with *de novo* AML, 3 had secondary AML from prior MDS, and 1 had high-risk MDS only. Although most participants had relapsed disease, eight had relapsed disease after at least one allogeneic stem cell transplant (SCT), five had relapsed disease despite induction chemotherapy but were either not candidates for SCT or they declined the intervention, and one had refractory disease.

**Table 1.** Participant Demographics and Clinical Characteristics (*N* = 14)

Variables	Mean (range, standard deviation)/***N*** (%)
Age	66 (35–85, 12.7)
Gender	
Male	11 (79%)
Female	3 (21%)
Race	
White	13 (93%)
African American	1 (7%)
Ethnicity	
Non-Hispanic/Latino	12 (86%)
Hispanic/Latino	1 (7%)
Preferred not to answer	1 (7%)
Marital status	
Married	13 (93%)
Divorced	1 (7%)
Education	
College degree	6 (43%)
Doctorate	4 (29%)
High school graduate	4 (29%)
Diagnosis (at time of interview)	
Acute myeloid leukemia	13 (93%)
Myelodysplastic syndrome	1 (7%)
Time from diagnosis to interview	
6–12 months	4 (29%)
1–2 years	7 (50%)
>2 years	3 (21%)
Received allogeneic hemopoietic stem cell transplantation	
Yes	8 (57%)
No	6 (43%)
Died within 3 months from interview	
Yes	7 (50%)
No	7 (50%)

One patient was interviewed in person and 13 patients were interviewed through video. At the time of analysis, 7 out of 14 participants died and all 7 died within three months of being interviewed. The following general themes were similar among participants of different age groups and transplant candidacy status.

**FIG. 2.**
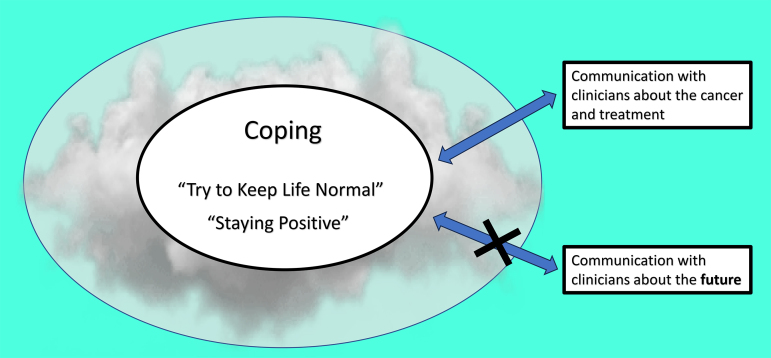
“Taking one day at a time” in a fog of uncertainty.

The overarching theme that explains participants' experiences living with R/R AML or high-risk MDS is *“Taking One Day at a Time” in a Fog of Uncertainty* (Fig. 2). Coping and patient–clinician communication were intrinsically linked to uncertainty.

### Uncertainty

Uncertainty dominated the experience due to the unpredictability of potential treatment outcomes, treatment side effects, and prognosis in this population. Participants portrayed uncertainty with these illustrative quotes:

*We're in uncharted territory.*

*It's too wide open.*

*The uncertainty is tough… whether anything is going to work.*

In the context of honest and open communication, several participants described doctors as simply not knowing all the answers and acknowledged their inability to predict the future.

*Nobody knows…doctors don't know, I don't know…what's the future.*

*Some things are clear cut, others are not…[such as] transplant…[I] did not see that as a guarantee.*

### Coping

Strategies for coping with the illness and its uncertainty included focusing on the present and trying to enjoy each day, maintaining normality in everyday life, distraction and keeping busy, staying positive, support from family and friends, and avoiding thinking about the worst-case scenario.

*Focus on today… what is going on in the moment.*

*I don't know what is going to happen, but I am not going to sit and dwell on something I can't change.*

*Live life the way we do now (if things worsen)*—*I don't think it would change much.*

*Try to keep life as normal as possible and find distractions… things that you enjoy doing.*

*I distract myself. I've been reading… sometimes puzzles. Just try to keep my mind busy in a useful way.*

*Try to walk, spend more time with my wife.*

*Family—friends—all so important*.

In contrast to the more positive coping responses, several participants described distress and discouragement as they shifted from early optimism to the reality of dying.

*Just trying to cope with it, when someone tells you that you have a certain shelf life, it's a real gut punch… at the same time not losing hope.*

*I feel stressed because I don't know what is going to happen… I may not make it… I may die.*

*I was riding the transplant train and being optimistic… things started going south… not traveling in a straight line… definitely more stressful.*

### Communication with clinicians

Patient–clinician communication about treatment options and potential outcomes was highly valued and participants reported that information was clear, honest, complete, and understandable. This positive communication exchange helped participants feel supported and helped them cope with uncertainty. Participants also noted the added value that nurses provided in reexplaining and reinforcing information that improved their understanding.

*Options were thoroughly explained.*

*I feel like [my doctors] are honest with me.*

*The nurses we have are quite amazing… she was always in constant communication with us.*

Recognizing the factual realities and uncertainty of treatment outcomes, some participants reported the importance of encouragement and a positive attitude from their clinicians.

*All patients… need to have encouragement.*

*You need to have a positive atmosphere around you.*

However, a few participants described receiving optimistic information early on and then being distressed later when cure was less likely. This suggests a desire for clinicians to balance optimism with the possibility of poor outcomes, given the uncertainty.

*I wish they hadn't pumped me up with optimism before they knew all the facts.*

*Raised my hopes and then my hopes got dashed.*

In contrast to communication related to treatment options, most participants were not ready or willing to discuss with their clinicians about the future, especially when their cancer worsens.

*When you come to that bridge, you cross it.*

*I think [discussing the future] would be helpful when it happens, but not before.*

*That would be a future discussion if things don't work… it's not where I need to focus my energy at the moment.*

These perspectives were influenced by patients and clinicians' prognostic expectations and/or minimal communication about prognosis. For example, only three participants recalled being told a specific timeframe for their prognosis, two of whom were told they have less than three months to live.

*I don't think we've reached the point where it was important to have those discussions.*

*Because they are optimistic, they're not ready to have that discussion with us.*

*This is something they're telling me is curable… as long as we try something different… they're not telling me I have a year or six months to live…*

These perspectives could also be influenced by preconceived perceptions about physician attitudes and expectations.

*They want their patients to be optimistic and have good attitudes.*

*They probably assume you're willing to do just about anything.*

*No one wants to have those conversations… on either side.*

Several participants were comfortable discussing the worst-case scenario with family.

*It gets you thinking… it lets your family know how you feel.*

*It reaffirms my own thinking… and it communicates [that information] with my caregivers.*

## Discussion

We explored the perspectives of patients with R/R AML and high-risk MDS on living with their illness and on discussing prognosis and the future with their clinicians. We found that most participants preferred living in the present, maintaining normality, and communicating with their clinicians about their illness and treatment options, as a means to cope with uncertainty. Most were not ready to discuss prognosis or their values and preferences in the event of worsening health with their clinicians until treatment was no longer viable and their life expectancy was short.

Prior qualitative studies in patients with hematological malignancies (HMs) have shown similar results. Booker et al. interviewed patients undergoing SCT and showed that although they agreed that advance care planning (ACP) was an important part of health care, the majority preferred staying positive, focusing on cure, and trying to survive their treatment experience.^20^ In another study including older patients with AML and MDS, patients reported that ACP discussions were emotional conversations, they feared knowing too much about their disease severity, and they tried to use optimism and hope to balance their fears.^21^ Patients did, however, value the parts of ACP discussions that improved disease understanding, minimized medical decision-making burden on their loved ones, and that allowed them to share what is meaningful to them with their clinicians. In a third study, also including older patients with AML and MDS, patients who were introduced to a structured serious illness communication tool felt that this communication approach was beneficial to their care, but wanted their clinicians to use positive language without taking away hope when discussing prognosis, even when the prognosis was poor.^10^

The findings from our study and prior research confirm that patients with HMs value patient–clinician communication to improve understanding about their illness and treatment options and value positivity and hope to maintain their emotional well-being. However, preferences for discussing more sensitive topics such as prognosis and the possibility of worsening health, with whom they prefer to discuss this with, and when to discuss, vary among individual patients.^9^ Although some want honest, open disclosure of information, such as prognosis, others do not want their clinicians to be specific about potential outcomes and will even explicitly tell clinicians not to take away their hope.

Research shows that clinician prognostic disclosure and patient prognostic awareness are low in patients with advanced HMs, and patients often overestimate their chances for cure.^2,9,22^ Unlike incurable solid tumors, HMs often have unpredictable disease trajectories ranging from unexpected health deterioration for some to the possibility of cure for others, making prognostication difficult for clinicians and patients. In addition, clinicians have difficulty communicating prognosis due to fears of taking away hope, lack of sufficient time, and feeling less comfortable discussing death and dying.^4,5^

Although many oncologists self-report discussing prognosis with their patients at diagnosis, only about one in six oncologists self-report discussing prognosis later in the disease trajectory.^23^ In our study, despite 50% of participants dying within three months of being interviewed, only three participants recalled being told a specific timeframe for their prognosis. It is possible that low prognostic awareness and high prognostic uncertainty in our study participants shaped their desire shaped their desire to focus on the present and avoid discussing the future with their clinicians.

Coping strategies such as living in the present and maintaining normality amid uncertainty could be viewed as avoidant coping, which involves withdrawing from stressors such as the thought of dying. Patients with solid tumors and high uncertainty have similarly shown increased use of avoidant coping strategies.^24,25^ However, the coping strategies of participants in our study could also be seen as positive and adaptive. A concept analysis of “Living in the Moment” for patients with life-limiting illnesses suggested that enjoying simple pleasures, prioritizing relationships, living each day to the fullest, maintaining normality, and not worrying about the future could all improve personal growth and preserve dignity despite uncertainty and the threat of dying.^26^

### Strengths and limitations

Strengths of this study include investigation of a very challenging communication gap in clinical practice between clinicians and patients with late-stage HMs. One limitation is that only one participant was diagnosed with MDS and further research with that population is warranted. Another limitation was related to the study purpose to interview patients, which eliminated caregiver perspectives, yet as some caregivers were present during the interview, we learned how critical caregivers were in understanding information, supporting the patient, and as a partner in day-to-day living.

### Implications for practice and future research

Although most participants in this study were not ready to discuss prognosis and their values and preferences for future care with their clinicians, it is still possible that patients with AML and high-risk MDS could benefit from these conversations earlier in the disease course. Clinicians need to carefully think about “who, what, when, and why.” Which patients and patient–caregiver dyads would benefit the most from these conversations? Which clinician should initiate the conversation? Should other members of the health care team be involved, such as social workers, chaplains, etc.? What topics would be most appropriate to discuss and when? This process should be repeated iteratively over the illness trajectory, as preferences for discussing certain topics change over time.

Future research on patient–caregiver dyads in this patient population would enhance our understanding of this populations' lived experience and inform strategies for patient–clinician communication.^27,28^ In our study, it was very clear that the perspectives of “taking one day at a time” and maintaining normality were a shared one between the caregiver or family of the participant. Family was also perceived as a strong support at clinical visits as described as “more brains thinking about the information you are hearing.” Additional research should also include developing and validating prognostic prediction models, optimizing clinician delivery of prognostic information despite prognostic uncertainty, and enhancing prognostic understanding for patients and their families.^9^

As we continue to study and implement early primary and specialty palliative care into clinical practice for this patient population, we should strive to identify novel communication interventions to help patients emotionally cope with uncertainty, support their desires to remain positive by providing encouragement and preserving hope, and assist patients to accomplish their goals for “taking one day at a time” and “keeping life normal.” These skills may be as crucial to discussing the future and preparing for the end of life.

Communication strategies such as identifying what can be done for the patient, focusing on immediate milestones and hurdles, emphasizing positive, but realistic treatment goals, and discussing ways to optimize quality of life in day-to-day living have been reported in the literature.^10,29^ Others include emphasizing what patients can control in their uncertain situation, identifying suitable coping strategies to deal with uncertainty, and emphasizing continued involvement in their care.^30^

## Conclusions

Patients with R/R AML and high-risk MDS are a unique population who experience unpredictable disease trajectories, high prognostic uncertainty, and high risk for dying. The participants interviewed in this study preferred living in the present, maintaining normality, and communicating with their clinicians about their illness and treatment options, as a means to cope with uncertainty. They were not ready or willing to discuss prognosis and the future with their clinicians. Additional research is needed to further understand patient and patient–caregiver dyad perspectives as a foundation to tailor and improve patient–clinician communication strategies.

## Abbreviations Used

ACP: advance care planning
AML: acute myeloid leukemia
HMs: hematological malignancies
MDS: myelodysplastic syndrome
R/R: relapsed and refractory
SCT: stem cell transplant
